# Sporadic Creutzfeldt-Jakob disease with unusual initial presentation as posterior reversible encephalopathy syndrome: a case report

**DOI:** 10.1186/s12883-016-0751-8

**Published:** 2016-11-22

**Authors:** Edgaras Dirzius, Renata Balnyte, Vesta Steibliene, Rymante Gleizniene, Inga Gudinaviciene, Andrius Radziunas, Kestutis Petrikonis

**Affiliations:** 1 Department of Psychiatry, Lithuanian University of Health Sciences, Mickeviciaus str. 9, Kaunas, LT-44307 Lithuania; 2 Department of Neurology Lithuanian University of Health Sciences, Mickeviciaus str. 9, Kaunas, LT-44307 Lithuania; 3 Department of Radiology Lithuanian University of Health Sciences, Mickeviciaus str. 9, Kaunas, LT-44307 Lithuania; 4 Department of Pathology Lithuanian University of Health Sciences, Mickeviciaus str. 9, Kaunas, LT-44307 Lithuania; 5 Department of Neurosurgery Lithuanian University of Health Sciences, Mickeviciaus str. 9, Kaunas, LT-44307 Lithuania

**Keywords:** Sporadic Creutzfeldt-Jakob disease, Posterior reversible encephalopathy syndrome, Magnetic resonance imaging, Biopsy, Case report

## Abstract

**Background:**

Creutzfeldt - Jakob disease (CJD) is a rapidly progressive and fatal neurodegenerative prion disease. MRI findings are included in diagnostic criteria for probable CJD, giving a sensitivity and specificity more than 90%, but the atypical radiological presentations in the early stage of the disease could cause the diagnostic difficulties. CJD can be definitively diagnosed by histopathological confirmation, brain biopsy or at autopsy.

**Case presentation:**

We present a case of 53-year-old woman with a history of a rapidly progressive dementia with symptoms of visual impairment, increased extrapyramidal type muscle tonus, stereotypical movements and ataxic gait resulting in the patient’s death after13 months. The clinical symptoms deteriorated progressively to myoclonus and akinetic mutism already on the 14th week. The series of diagnostic examinations were done to exclude the possible causes of dementia. Initial MRI evaluation as posterior reversible encephalopathy syndrome (PRES) on the 9th week after the onset of symptoms created us a diagnostic conundrum. Subsequent MRI findings of symmetrical lesions in the basal ganglia (nucleus caudatus, putamen) on the 13th week and EEG with periodic sharp wave complexes (PSWC) in frontal regions on the 18th week allowed us to diagnose the probable sCJD. The histopathological findings after brain biopsy on the 14th week demonstrated the presence of the abnormal prion protein deposits in the grey matter by immunohistochemistry with ICSM35, KG9 and 12 F10 antibodies and confirmed the diagnosis of sCJD.

**Conclusions:**

In this article we focus our attention on a rare association between radiological PRES syndrome and early clinical stage of sCJD. Although concurrent manifestation of these conditions can be accidental, but the immunogenic or neuropeptide mechanisms could explain such radiological MRI findings. A thorough knowledge of differential diagnostic of PRES may be especially useful in earlier diagnosis of sCJD.

## Background

Creutzfeldt - Jakob disease (CJD) is a rapidly progressive and fatal neurodegenerative prion disease, known as the human transmissible spongiform encephalopathy [1]. Worldwide, an estimated incidence of CJD is 1–2 cases per 1 million people a year. Three categories of CJD are described: sporadic, familial and acquired [2, 3]. The most frequent is sporadic CJD (sCJD), with absence of known risk factors for the disease, and this category accounts for about 85% of all cases. The mean age at disease onset is about 64 years [4]; and the mean duration of the disease is approximately 8 months. Patients with CJD typically present with a rapidly progressive dementia, cerebellar dysfunction, visual abnormalities, pyramidal and extrapyramidal symptoms. Myoclonus is almost the one constant physical sign in about 90% of cases, although its manifestation varies depending on genetic background and disease stage [5–7]. Such conditions like confusion, agitation, visual hallucinations or depression could be psychiatric early stage sCJD symptoms. In terminal stages of the disease, typically a severe dementia and akinetic mutism develop. Supporting tests include the presence of 14–3–3 protein in the cerebrospinal fluid (CSF) and periodic sharp wave complexes (PSWCs) in EEG [8–12]. Such magnetic resonance imaging (MRI) abnormalities, as alterations on diffusion weighted images (DWI) or fluid attenuated inversion recovery images (FLAIR) in caudate nucleus and/or putamen and in at least two cortical regions (temporal, parietal or occipital) have been added to diagnostic criteria for probable CJD, giving a sensitivity and specificity more than 90%. However, CJD can only be definitively diagnosed by histopathological confirmation, usually brain biopsy or at autopsy [13, 14].

According to the incidence of CJD in general population, since population of Lithuania is 2 872 294 people, there should be 2–3 cases of CJD per year occurring in Lithuania [1, 5, 15]. Nevertheless, since 2000, only 4 cases of sCJD have been reported - all of them were diagnosed post-mortem. We could assume that due to a lack of experience in diagnosing CJD in Lithuania, the disease is probably misdiagnosed [16]. Despite the typical symptoms of CJD in the disease progression, the most challenging aspect of making right diagnosis in early stages is interpretation of MRI findings, which can cause diagnostic difficulties [17–19]. In particular, when in the Heidenhain variant of sCJD the patients had isolated visual symptoms, but MRI findings at the disease onset were normal [20]. In other cases, during early disease stages MRI showed only the diffusion restriction pattern in the parieto-occipital cortex, with no other changes typical for CJD [17]. It was shown that different clinico-pathological variant sCJD has different PrP^Sc^ variants. Recently it was found out that Heidenhain variant of sCJD is usually associated with codon 129MM genotype and prevalent PrP^Sc^ type 1 [21].

Following the CARE guidelines [22], we describe the clinical presentation of a recent case of sCJD in Lithuania and present the experienced diagnostic difficulties, when MRI changes in sCJD initially presented as Posterior reversible encephalopathy syndrome (PRES).

## Case presentation

**Fig. 1:**
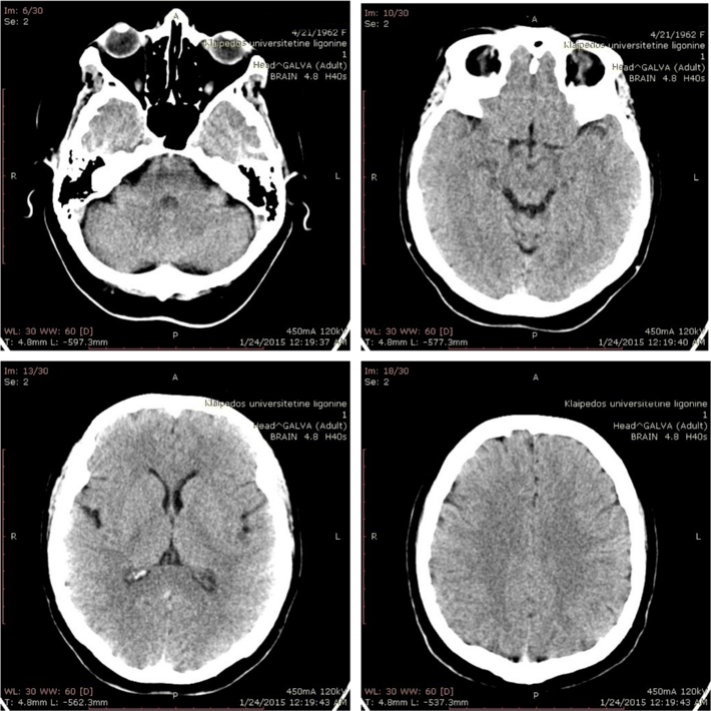
Brain computer tomography without contrast at week 2. Legend: no evidence of lesions

**Fig. 2:**
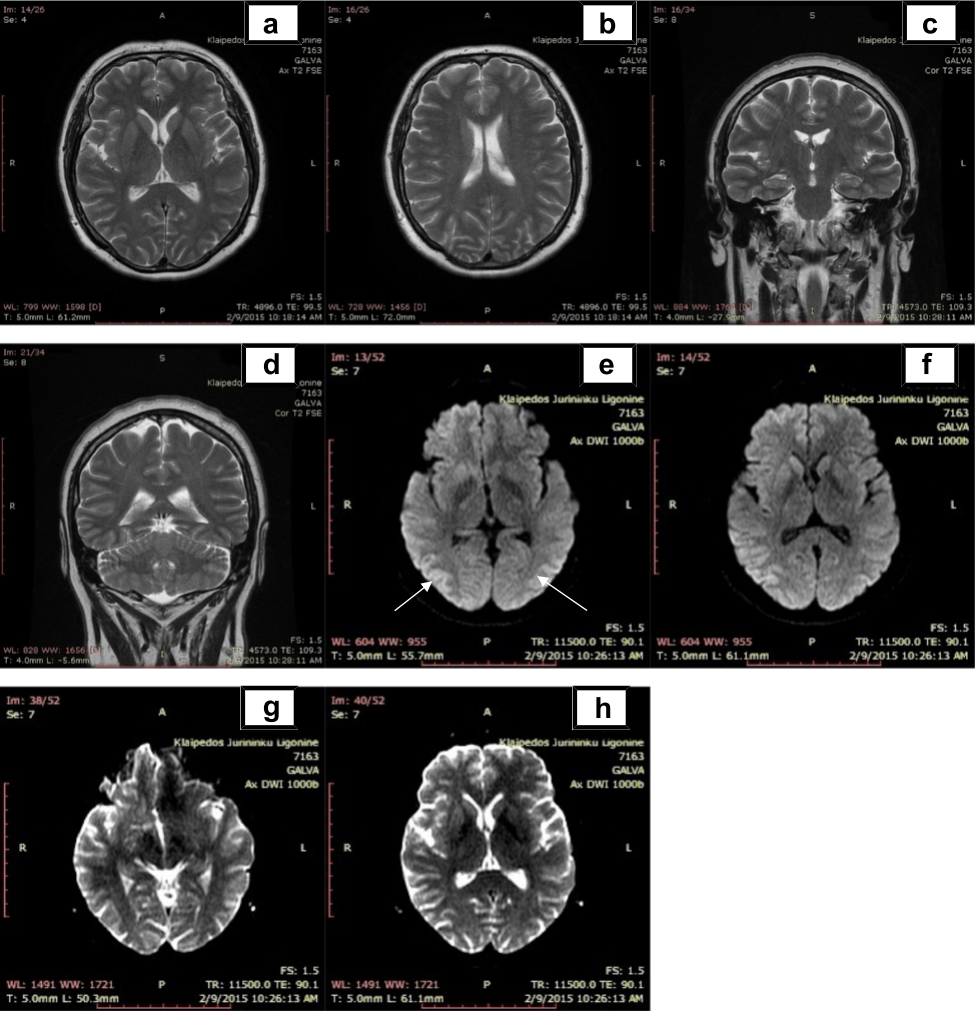
Brain magnetic resonance images at week 2. Legend: the primary evaluation – no significant changes. Retrospective reevaluation - slight increase in signal intensity in the occipital cortex DW sequence (**e**, **f**), without pathological lesions in the other sequences (**a**, **b**, **c**, **d**, **g** and **h**)

**Fig. 3:**
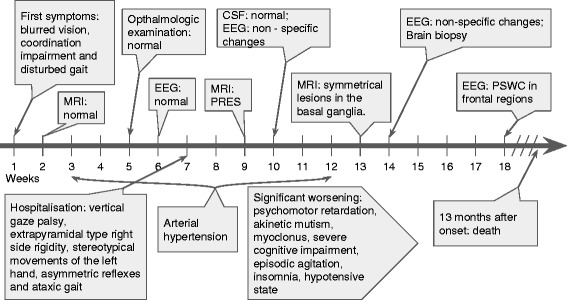
Timeline of case report

The disease manifested in a healthy 53 years old female patient 7 weeks prior to the initial visit to our center with sudden onset of blurred vision, dizziness, disturbed gait and coordination impairment. Before the manifestation of symptoms patient was healthy, no prior dementia cases in patient’s family history were recorded. Two weeks after initial symptoms presentation patient was hospitalized in the Department of Neurology of Regional Hospital. Neither ophthalmologic examination nor blood test revealed any significant changes. Brain computed tomography (CT) (Fig. 1) and MRI (Fig. 2) were evaluated as normal. Although after retrospective reevaluation of MRI, slight increase in the occipital DW signal was found (Fig. 2). EEG was performed on the 6th week since initial disease presentation did not show any specific changes. Initially, the patient was diagnosed with primary hypertension (blood pressure was 150/70 mmHg): Spironolactone 25 mg per day for arterial hypertension and Clonazepam 0,5 mg once a day for insomnia, Betahistine 3 mg three times a day were prescribed. On the sixth week after initial symptoms due to complaints of dizziness, impaired memory, insomnia, emotional lability, the possible causes were differentiated among cerebrovascular and somatoform/conversion disorder. As the symptoms progressed significantly, on the 7th week after the onset of symptoms, the patient was referred to the University Hospital. Clinical course of the disease and the major diagnostic tests are presented in Fig. 3.

During the hospital admission the patient complained of weakness, difficulties standing up and walking due to dizziness and visual impairment as well as difficulties concentrating during the interview. A neurological evaluation revealed vertical gaze palsy, extrapyramidal type increased right body side muscle tonus, involuntary stereotypical movements (purposeless raising and lowering of the left hand), weaker reflexes on the left side and ataxic gait. Arterial blood pressure was 160/90 mmHg. Ophthalmologic examination revealed severely impaired vision, a disability to distinguish between light and darkness, without any congestive changes in the retina. The evaluation of mental state revealed typical symptoms of organic brain disease: disorientation in time, slower thought processing, concentration difficulties and disturbed short-term memory. Mini-Mental State Examination score 20/30 revealed dementia with moderate cognitive decline, two in date and two points in place orientation were missed, three in recall, two in attention and calculation, one in repetition. Due to episodic anxiety, agitation and fearful gaze, visual hallucinations were suspected. Physical examination of other systems did not reveal any significant abnormalities.

During the next two weeks blood tests were carried out to rule out infectious, endocrine and rheumatologic diseases, metabolic conditions and secondary autoimmune central nervous system vasculitis since they are common causes of dementia; genetic testing, urine analysis and a liver biopsy were performed in search for Wilson’s disease, but all the tests were negative. Standard examination of CSF did not reveal significant deviations from the normal range. Despite antihypertensive treatment, arterial blood pressure was ranging between 160/100 and 140/85 mmHg.

**Fig. 4:**
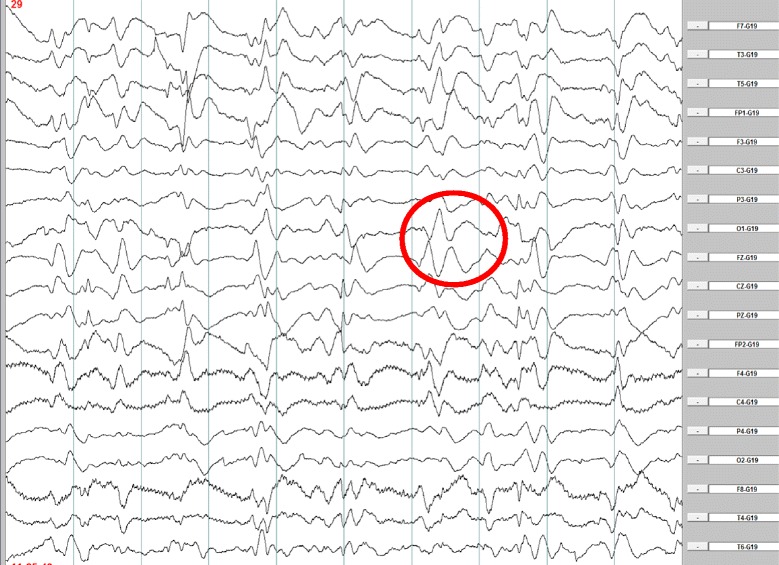
Electroencephalography at week 18. Legend: periodic sharp wave complexes in frontal regions

EEGs performed on the 10th and 14th weeks after the onset of symptoms showed non-specific diffuse slowing activity with rhythmic delta activity in frontal brain regions (FIRDA), predominantly in the left side. Only at the 18th week EEG showed periodic (repeated every 1 s) sharp wave complexes (PSWC) in frontal regions (Fig. 4).

**Fig. 5:**
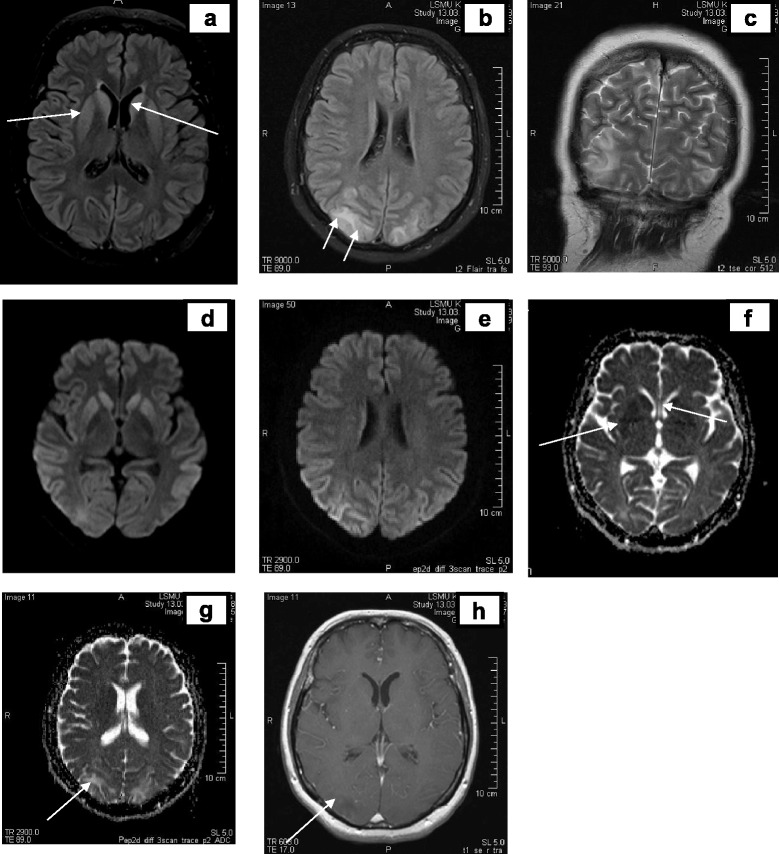
Magnetic resonance images at week 9. Legend: hyperintense lesions in T2W/FLAIR, T2W, DW, ADC (**b**, **c**, **d**, **e**, **g**) sequences in the occipital lobes cortex (*both sides*) and subcortical white matter (*in the right side*) *arrows* slight hiperintense lesions in bilateral subcortical nuclei (head of the *nucleus caudatus, n. lentiformis*) in T2W/FLAIR, DW (**a**, **d**, *arrows*) sequences with hypointensive signal SI lesions in ADC coefficient (**f**); hypointense lesion in the right occipital lobe in T1W sequence (**h**, *arrow*) with some contrast media enhancement dots

The MRI, done on the 9th week (Fig. 5) showed in T2W increased signal intensity (SI) zones in cortical gray and subcortical white matter, especially on the right side without restriction in DWI sequence, in the parietal-occipital area supplied by the posterior cerebral artery, leading to the conclusion of posterior reversible encephalopathy syndrome (PRES). The typical imaging finding is vasogenic oedema in the subcortical white matter of the parietal and occipital lobes [23]. As an experience with PRES grows, atypical presentations of PRES are being increasingly described: the cases with atypical vasogenic oedema patterns of distribution, such as frontal lobe, cerebellum, basal ganglia or brain stem involvement [23–26]. This non-specific radiological pattern in our case also raised a new diagnostic challenge. Normalization of blood pressure due to antihypertensive treatment and other symptomatic treatment did not improve neurologic symptoms. During the period of the next two weeks all possible causes (vascular, ictal, infectious) of PRES were ruled out [27]. A diagnosis of CJD was suspected the first time.

**Fig. 6:**
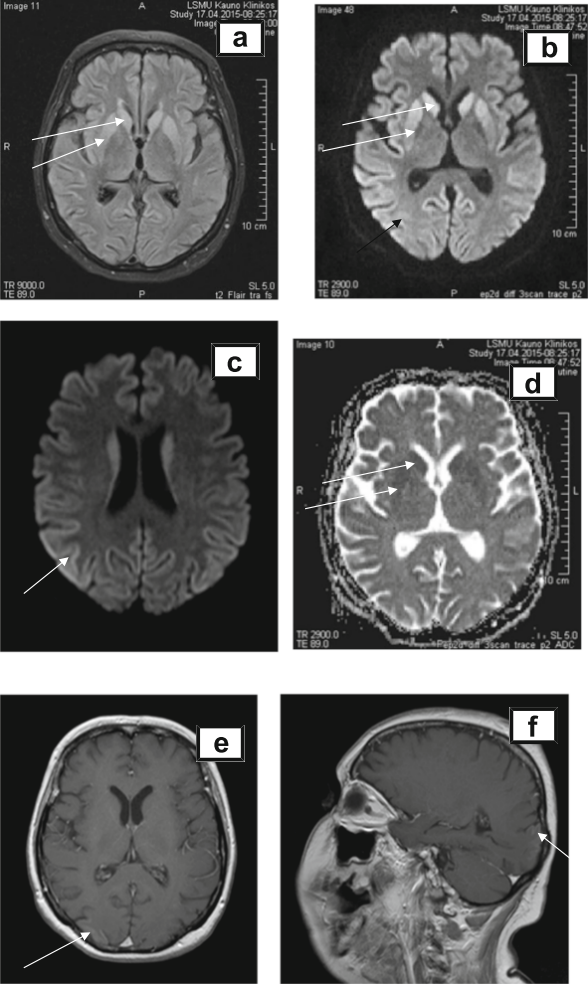
Magnetic resonance images at week 13. Legend: hyperintense lesions in the basal ganglia (head of *nucleus caudatus, nucleus lentiformis,* arrows) in T2W, DW sequences (**a**, **b**, **c**, **d** *arrows*), residual cortical ribboning (hyperintense lesions in DW sequence **b**, **c**, *arrows*) and cortical contrast enhancement in T1W sequence (**e** axial, **f** sagittal planes, *arrows*)

In MRI, repeated after four weeks, on the 13th week (Fig. 6) symmetrical lesions in the basal ganglia (head of *nucleus caudatus, putamen*) were found. The previous lesions in the cortical/subcortical area were absorbed. The lesions found in the basal ganglia led us to suspect CJD or extrapontine myelinolysis [10, 28].

Within 14th week of the disease onset and the symptomatic treatment, the patient’s condition significantly deteriorated. Severe psychomotor retardation with hyper tonus of neck and arms flexor muscles, jerky myoclonic movements, repeated episodes of agitation and severe insomnia were observed. Condition progressed to akinetic mutism with severe cognitive impairment. The blood pressure reversed into hypotensive state. There was no possibility in Lithuania to test the 14–3–3 protein, most widely used CSF biomarker for CJD and one of the WHO criteria, for probable CJD. Taken into account a rapidly progressive dementia, clinical manifestation of myoclonus, visual changes, ataxia, muscle hyper tonus, akinetic mutism, symmetrical MRI findings of basal ganglia, on the 14th week of the disease the decision to take brain biopsy was made.

**Fig. 7:**
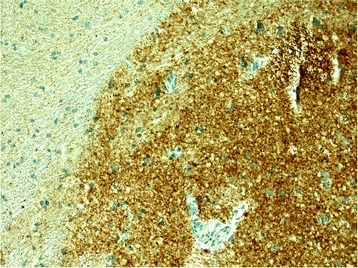
Immunostaining for the abnormal prion protein. Legend: diffuse strong synaptic labelling in all the grey matter regions (12 F, KG9, ICSM35)

The brain tissue biopsy from occipital brain lobe and head of caudate nucleus was performed. Histological evaluation was performed in the Division of Neuropathology of the National Hospital for Neurology and Neurosurgery, Queen Square, London: the abnormal prion protein deposits (detected with antibodies ICSM35, KG9 and 12 F10) were seen in all the grey matter areas (Fig. 7). No specific features in small biopsy samples were found to suggest iatrogenic or inherited forms of prion disease. Patient had not received any past treatment with human cadaver derived growth hormone, undergone neurosurgery with human cadaver derived dural graft or scleral transplant, patient had not received blood transfusion, which suggests a possible sporadic case of CJD. The neuropil in the grey matter of caudate nucleus showed mild but widespread micro-vacuolar degeneration. In the neuropil of occipital cortex mild micro-vacuolar degeneration is patchy. Immunostaining for the abnormal prion protein revealed diffuse strong synaptic labelling in all the grey matter regions. In the white matter there are freaquent granular deposits but no convincing filamentous labelling. Histopathological and immuno-histochemical findings of prion protein (scrapie) (PrP^Sc^) and summarizing all the data, we confirmed prion disease, compatible with sCJD. The patient eventually died 13 months after disease onset. Autopsy was not carried out.

## Conclusion

To our knowledge, there were studies reporting white mater signal alterations in MRI [21], but this is one of few through case reports where radiological presentation of PRES was the initial MRI finding in an early stage of the disease [29, 30]. PRES is a clinic-radiological syndrome precipitated by various diseases and conditions and has evolved with increasing availability of MRI. Generally, MRI reveals posterior white matter oedema due to vascular cerebral dysregulation, ordinarily symmetrical in the parietal and occipital lobes. The most common clinical manifestations of PRES are seizures, headache, encephalopathy, visual changes and vertigo. Usually hypertension is highlighted as a regular trait of all PRES associated conditions [29, 31, 32]. Kidney disease, autoimmune disease, malignancy, organ transplant, cytotoxic medication, renal artery stenosis, sepsis, pre/eclampsia, Takayasu’s arteritis, Sheehan syndrome and multi-organ dysfunction are the most common in other PRES-associated conditions [26]. When treatment of underlying condition is initiated, PRES symptoms generally diminish within few days [33, 34]. Primary central nervous system (CNS) vasculitis, referred as primary angiitis of the CNS (PACNS) was the one of PRES differential diagnostic conditions that could lead to such unexplained neurologic deficit. No possible causes for the secondary CNS vasculitis were found. The absence of specific serological tests and CSF markers have interfered the confirmation of PACNS diagnosis. Only a brain biopsy is the gold standard test, so this diagnostic challenge remained until the biopsy results were obtained [35].

Despite the fact that CJD is included into the differential diagnostics of PRES, it is quite unusual disease presentation [19]. The primary radiological presentation of CJD gave us a conundrum in the pathophysiology of the disease. Four main theories on the pathogenesis of PRES are known: vasogenic, cytotoxic, immunogenic and neuropeptide [29]. In our case, the primary arterial hypertension was considered as possible causative factor for manifestation of PRES symptoms. Otherwise, 15–20% of PRES cases were normotensive and blood pressure variations in PRES group did not differ from matched controls [36]. However, antihypertensive treatment did not reduce the expression of symptoms and vasogenic theory insufficiently explained the abundance of neurological symptoms. In some cases, PRES was explained by cytotoxic theory, in association with toxins or chemokines, either from endogenous or exogenous synthesis, which leads to endothelial dysfunction and cerebral oedema [23, 29, 37]. A slow progression of clinical symptoms in our case and no significant infection or other probable source of cytotoxins let us to exclude the cytotoxic pathogenesis of PRES.

Immunogenic and neuropeptide theories of PRES are intertwined: chemokines, cytokines and vasoconstrictors dysregulation are involved; it leads to vasospasm, ischemia and possible manifestation of PRES [29]. There is the evidence, that immunization with some types of CJD prion proteins can induce activation of microglia and dysfunction of vasoconstrictors upregulation [38, 39]. Therefore, immunogenic or neuropeptide theories could explain the pathogenesis of initial manifestation of PRES in our patient.

However, such atypical MRI presentation of CJD has led to the delay of probable CJD diagnosis. When patient was admitted to our hospital we only had written conclusion of MRI investigation which concluded that no significant changes were found. Although after retrospective reevaluation of initial MRI we noticed slight occipital signal intensity changes, in early clinical stages misdiagnoses are not uncommon [21, 30, 40]. The other diagnostic limitation in the early stage of the disease was that the 14–3–3 protein, CSF biomarker for CJD, was not tested [14]. On the other hand, the 14–3–3 protein is known to be non-specific for CJD. The immuno-histochemical findings of PrP^Sc^ in the brain tissue gave a definite confirmation of sCJD [8].

Also it should be noted that PRES could be caused by various other conditions, including high blood pressure [41]. Our patient had high blood pressure for some time and it is possible, that PRES manifestation with progression of sCJD was just a coincidence.

In this article we reported and focused our attention on a rare association between radiological PRES syndrome and early clinical stage of sCJD. Although we must emphasize that PRES and CJD co-manifestation could be accidental and there is a need of further extensive research to confirm or reject association, it seems that the immunogenic or neuropeptide mechanisms could explain the radiological findings presented in MRI of our patient. Thus, a thorough knowledge of differential diagnostic of PRES and clinical experience in early recognition of clinical symptoms of CJD may be especially useful in earlier diagnosis of sCJD.

## Abbreviations

CJD: Creutzfeldt-Jakob disease
CNS: Central nervous system
CSF: Cerebrospinal fluid
CT: Computed tomography
DWI: Diffusion weighted images
EEG: Electroencephalogram
FIRDA: Delta activity in frontal brain regions
FLAIR: Fluid attenuated inversion recovery images
MRI: Magnetic resonance imaging
PACNS: Primary angiitis of the central nervous system
PRES: Posterior reversible encephalopathy syndrome
PrP^Sc^: Prion protein (scrapie)
PSWC: Periodic sharp wave complexes
sCJD: Sporadic Creutzfeldt-Jakob disease
SI: Signal intensity
WHO: World Health Organization
